# Supplementary material for: Natural Contaminants in Wines: Determination of Biogenic Amines by Chromatographic Techniques
Source: Int J Environ Res Public Health. 2021 Sep 27;18(19):10159. doi: 10.3390/ijerph181910159 (PMC8508579; doi:10.3390/ijerph181910159)
Supplement: Supplementary file 1 [file ijerph-18-10159-s001.zip › ijerph-1346224_supplementary.pdf]

SUPPLEMENTARY

Article

Natural Contaminants in Wines: Determination of Biogenic Amines by Chromatographic Techniques

Giuliana Vinci *, Lucia Maddaloni, Sabrina A. Prencipe and Roberto Ruggieri

Department of Management, Sapienza University of Rome, Via del Castro Laurenziano 9, 00161 Rome, Italy; giuliana.vinci@uniroma1.it (G.V.); lucia.maddaloni@uniroma1.it (L. M.); sabrinaantonia.prencipe@uniroma1.it (S. A. P.); roberto.ruggieri@uniroma1.it (R.R.).

* Correspondence: giuliana.vinci@uniroma1.it

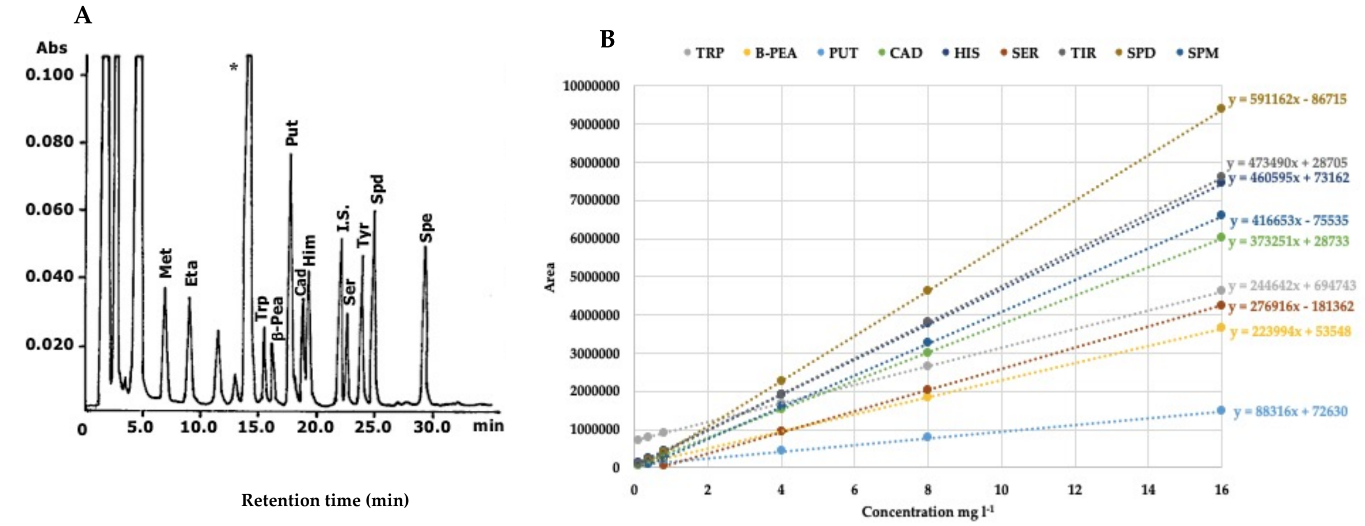

Figure S1. (A) Chromatogram of BAs standard solution (*1.7 diaminoheptane (IS)) (B) Calibration curves of nine BAs.

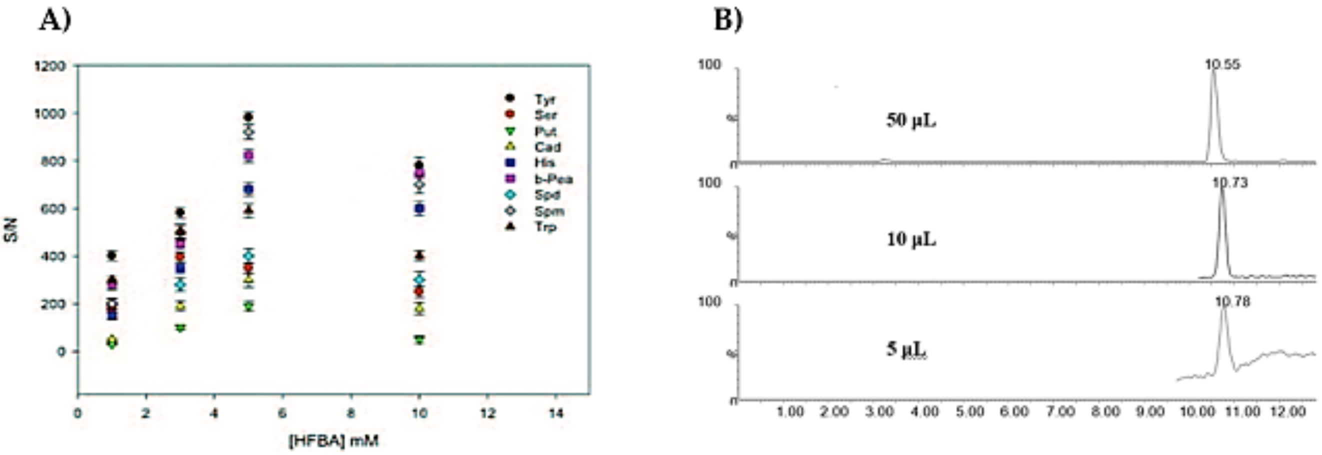

Figure S2. (A) Biogenic amines solution at four different HFBA concentrations, ranged from 1 mM to 10 mM; **(B)** Optimization of the volume of samples injected.

Table S1. Performance characteristics of HPLC-UV/Vis method.

Biogenic Amines	Conc. Range (mg l^{-1})	R^2	LIN (%)[1]	LOD (µg l^{-1})[2]	AS (mg l^{-1})[3]	R % (RSD)[4]
**TRP**	0.1 – 16.0	0.999	98.99	42.8	0.231	103 (3)
**ß-PEA**	0.1 – 16.0	0.998	99.20	64.2	0.373	99 (2)
**PUT**	0.1 – 16.0	1.000	99.70	8.0	0.033	101 (2)
**CAD**	0.1 – 16.0	0.999	99.25	17.1	0.082	100 (2)
**HIS**	0.1 – 16.0	0.999	99.85	50.4	0.271	101 (2)
**SER**	0.1 – 16.0	0.990	98.75	54.2	0.887	100 (2)
**TYR**	0.1 – 16.0	0.999	99.10	61.2	0.303	94 (2)
**SPD**	0.1 – 16.0	1.000	99.97	20.4	0.111	103 (3)
**SPM**	0.1 – 16.0	0.999	98.96	27.0	0.147	103 (2)

[1] LIN (%) = "on-line" linearity; [2] LOD = limit of detection; [3] AS = analytical sensitivity,

Table S2. Performance characteristics of the LC-ESI-MS method.

Biogenic Amine	Conc. range (mg l^{-1})	R^2	LIN %	AS (µg l^{-1})	LOD (µg l^{-1})
**Tyramine**	0.1 - 16	0.999	98.99	23.1	42.8
**ß-Phenylethylamine**	0.1 - 16	0.998	99.20	37.3	64.2
**Putrescine**	0.1 - 16	1.000	99.70	3.3	8.0
**Cadaverine**	0.1 - 16	0.999	99.25	8.2	17.1
**Histamine**	0.1 - 16	0.999	99.85	27.1	50.4
**Serotonin**	0.1 - 16	0.990	98.75	37.7	66.9
**Tryptamine**	0.1 - 16	0.999	99.10	30.3	61.2
**Spermidine**	0.1 - 16	1.000	99.97	11.1	20.4
**Spermine**	0.1 - 16	0.999	98.96	14.7	27.0

[1] LIN (%) = “on-line” linearity; [2] LOD = limit of detection; [3] AS = analytical sensitivity.

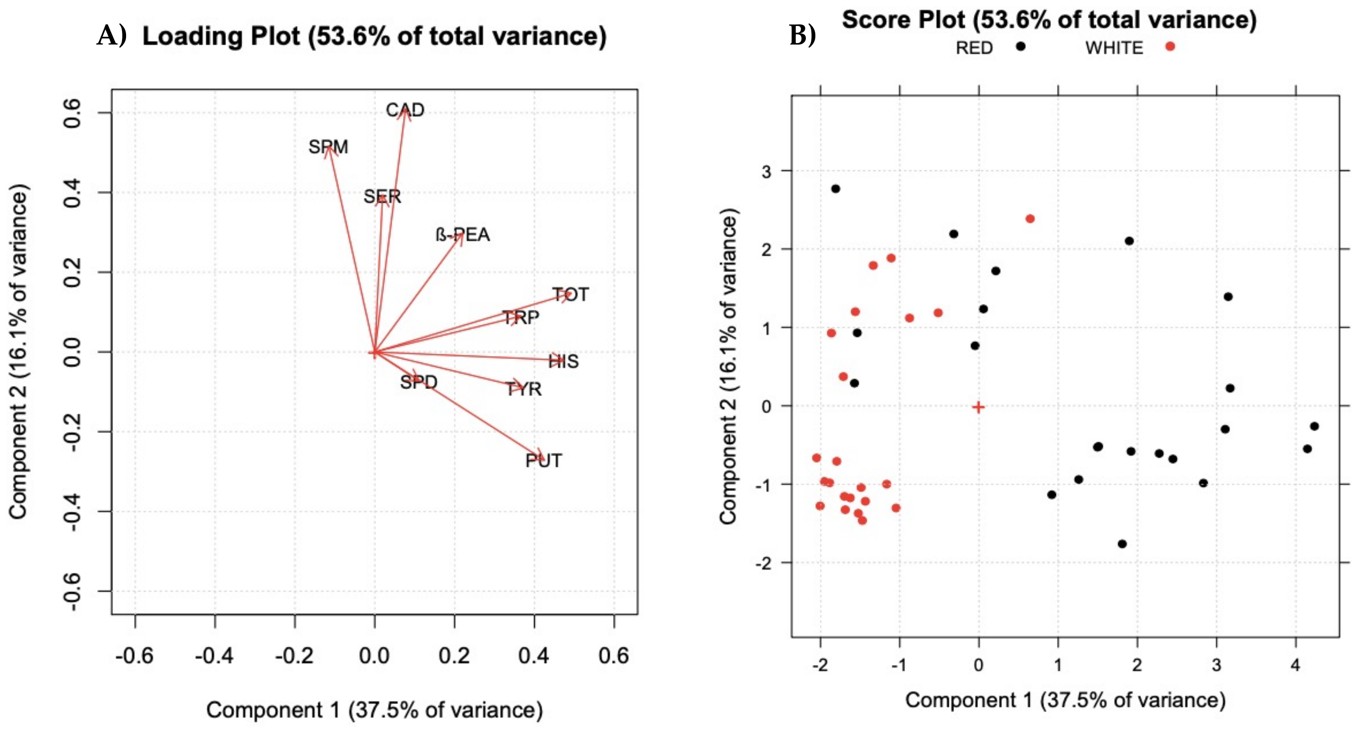

Figure S3. A) Loadings Plots of nine biogenic amines B) Scree Plot of 44 wine samples.
